# The Significance of True Knot of the Umbilical Cord in Long-Term Offspring Neurological Health

**DOI:** 10.3390/jcm10010123

**Published:** 2020-12-31

**Authors:** Yael Lichtman, Tamar Wainstock, Asnat Walfisch, Eyal Sheiner

**Affiliations:** 1 Department of Obstetrics and Gynecology, Soroka University Medical Center, Ben-Gurion University of the Negev, Beer-Sheva 8410501, Israel; 2 Department of Public Health, Faculty of Health Sciences, Ben-Gurion University of the Negev, Beer-Sheva 8410501, Israel; wainstoc@bgu.ac.il; 3 Department of Obstetrics and Gynecology, Hadassah Mt. Scopus Medical Center, Jerusalem 9112001, Israel; asnatwalfisch@yahoo.com

**Keywords:** true knot of cord, perinatal outcomes, long-term neurological morbidity, intrauterine hypoxia

## Abstract

We aimed to study both the short- and long-term neurological implications in offspring born with confirmed knotting of the umbilical cord—“true knot of cord”. In this population based cohort study, a comparison of perinatal outcome and long-term neurological hospitalizations was performed on the basis of presence or absence of true knot of cord. A Kaplan–Meier survival curve was constructed to compare the cumulative incidence of neurological hospitalizations between the study groups. Multivariable regression models were used to assess the independent association between true knot of cord, perinatal mortality and long term neurological related hospitalizations, while controlling for potential confounders. The study included 243,639 newborns, of them 1.1% (*n* = 2606) were diagnosed with true knot of the umbilical cord. Higher rates of intrauterine fetal demise (IUFD) were noted in the exposed group, a finding which remained significant in the multivariable generalized estimation equation, while controlling for confounders. The cumulative incidences of neurological hospitalizations over time were comparable between the groups. The Cox regression confirmed a lack of association between true knot of cord and total long term neurological related hospitalizations. While presence of true knot of the umbilical cord is associated with higher IUFD rates, in our population, however, its presence does not appear to impact the long term neurological health of exposed offspring.

## 1. Introduction

Knotting of the umbilical cord—“true knot of cord”—is a rather rare event (about 1% of term deliveries) [1] and a challenging antepartum diagnosis [2]. Factors predisposing the formation of cord knots include polyhydramnios and multi-parity (due to uterine laxity), as well as diabetes and preterm delivery, all of which enable exaggerated fetal movements [3,4,5]. Other factors that have been associated with true knots are male fetuses and long cords, probably due to the fact that these two often coexist [4,6,7]. The pathophysiology of knotting of the cord is probably a combination of uterine laxity, exaggerated fetal movement and increased amount of amniotic fluid relative to fetal size.

The exact timing of formation of these cord knots is a matter of debate—some argue that knotting of the umbilical cord takes place early in the antenatal course during the late first trimester due to increased amniotic fluid volume/fetal size ratio, while others think that this event mainly takes place during labor [8]. Attempts to diagnose this condition antepartum have been disappointing [2], even with the latest advancements in Doppler sonography, and most cases are recognized only postpartum [9].

The significance of true cord knot is controversial; while several studies show an association with devastating perinatal outcomes (such as intrauterine fetal demise (IUFD), meconium-stained amniotic fluid (MSAF), and low Apgar scores) [4,10,11,12], others have failed to establish any clinical significance [13,14]. Some studies link true cord knots to low birth weight [15], potentially resulting from chronic intrauterine hypoxia [16], while others show no such association [11]. This might be explained by the level of tightness of the knot, also affected by the protection of Wharton’s jelly [17].

Although immediate obstetrical outcomes related to cord knots have been extensively studied, much less is known regarding its long term significance. Our work aimed to shed light on the potential long term neurological impact of true cord knots in exposed offspring.

## 2. Experimental Section

This was a population based retrospective cohort study conducted at the Soroka University Medical Center (SUMC) between the years 1991–2014. SUMC is a sole tertiary medical center located in the Negev region of Israel, which spreads over 60% of Israel’s territory with a population of 730,000 inhabitants in 2017 (and constantly increasing) [18].

Currently, SUMC providing tertiary medical services to about 1,190,000 individuals (composing around 14% of Israel population). The study was approved by the institutional review board (SUMC IRB) and is based on nonselective population data.

The Bedouin Arabs of the Negev are a Muslim society [19]. Bedouin culture places great importance on family and high fertility is central in this society [20]. Thus, multiparity is common [21]. The available prenatal diagnostic services are underused by this population, possibly owing to religious restrictions [22,23], distrust of conventional medical care providers and facilities, geographical distance to healthcare services including available prenatal care services, and patriarchal restriction of female autonomy [19,24].

The primary exposure was presence of true knot of cord, as recorded postpartum by the midwife attending the delivery, in the computerized as well as paper perinatal records which are constantly being revised by professional hospital secretaries for error. The outcome measures included immediate obstetrical outcomes as well as any neurological related hospitalization of the offspring up to 18 years of age, as evident by any neurological diagnosis mentioned in the patients files upon admission to SUMC (for any reason). This was defined as having one diagnosis or more from a pre-defined list of ICD-9 neurological codes detailed in the Appendix A (Table A1). Multiple gestations and congenital malformations cases were excluded from all analyses. If a cord description was missing from the record, it was excluded from the analysis. Follow up time was defined as time to an event or censoring. An event was defined as hospitalization with a neurological diagnosis, including all the non-neurological admissions in which a neurological diagnosis (chronic or acute), was designated for the offspring. Censoring occurred either as death during any hospitalization (other than neurological related), end of study period (January 2014), or when the child reached 18 years of age (calculated according to date of birth). Only the first admission with a neurological related diagnosis for each child was included in the analyses.

Data were collected from two databases that were cross-linked and merged: the computerized hospitalization database of SUMC (“Demog-ICD9”), and the computerized perinatal database of the SUMC obstetrics and gynecology department. The Demog-ICD9 database includes demographic information and ICD-9 codes for all medical diagnoses made during hospitalizations at SUMC. The perinatal database consists of information recorded immediately following delivery by an obstetrician or a midwife. Experienced medical secretaries routinely review the information prior to entering it into the database to insure its maximal completeness and accuracy. Furthermore, the perinatal database was regularly tested and validated by the Department of Epidemiology, Ben-Gurion University of the Negev, Beer Sheva, Israel. Coding is performed after assessing medical prenatal care records as well as routine hospital documents.

Screening for neurological morbidity in the hospital setting is done in the Institute for Child Development which provides diagnostic services, treatment, and follow up, for children with developmental disorders up to age 6 years. The Institute for Child Development has close ties with other ambulatory services and as a consequence, even though the Institute for Child Development’s diagnoses are not part of the SUMC hospitalization database, they are often presented as background diagnoses of the child upon hospitalization. Early assessment of developmental difficulties and disorders occur in Israel routinely at community. If additional evaluation is needed, the children and their families are referred to Child and Family Developmental Centers, where the child is been evaluated. When a child previously diagnosed in a community clinic is being admitted to the hospital, his previous diagnoses are usually exported to the SUMC data base. Additionally, the community clinic and SUMC share the same online interface, which facilitates the process of exporting diagnoses upon admission [25].

Statistical analysis was performed using the SPSS package, 23rd edition (IBM/SPSS, Chicago, IL, USA). Differences in categorical data were assessed by chi-square for general association. T-test was used for comparison of continuous variables with normal distribution. Kaplan–Meier survival analysis was used to compare the cumulative incidence of neurological related hospitalizations over time, up to 18 years of age. A multivariable generalized estimation equation model was used to study the association between true knot of the cord and perinatal mortality. Cox proportional hazards analysis was used to assess a possible independent association between true knot of cord and long term neurological related hospitalizations of the offspring. Both multivariable models adjusted for potential confounding variables and clinically relevant characteristics. These included: gestational age, small for gestational age (SGA, <5th percentile of birthweight according to gestational age and gender), ethnicity, smoking status, maternal diabetes and hypertension. A *p* value of < 0.05 (two sided) was considered statistically significant.

## 3. Results, Figures and Tables

### Results

During the study period, 243,639 newborns met the inclusion criteria. Of them, 1.1% (*n* = 2606) were diagnosed with confirmed true knot of the umbilical cord. Maternal characteristics and pregnancy outcomes in both groups are shown in Table 1. Parturient with true knot of cord were significantly more likely to be multiparous, suffer from hypertension, diabetes, undergo labor induction, and deliver preterm (<37 0/7 weeks’ gestation). Deliveries were more likely to involve meconium stained amniotic fluid (MSAF), and to end with cesarean delivery. Newborns in the exposed group exhibited higher rates of low (<7) Apgar scores, SGA infants, and IUFD.

In the multivariate generalized estimating equation models an independent and significant association was found between presence of a true cord knot and IUFD, while adjusting for ethnicity, smoking status, maternal diabetes, maternal hypertension and offspring date of birth (adjusted odds ratio 3.606; 95% CI 2.685–4.841, *p* < 0.001; Table 2).

**Figure 1:**
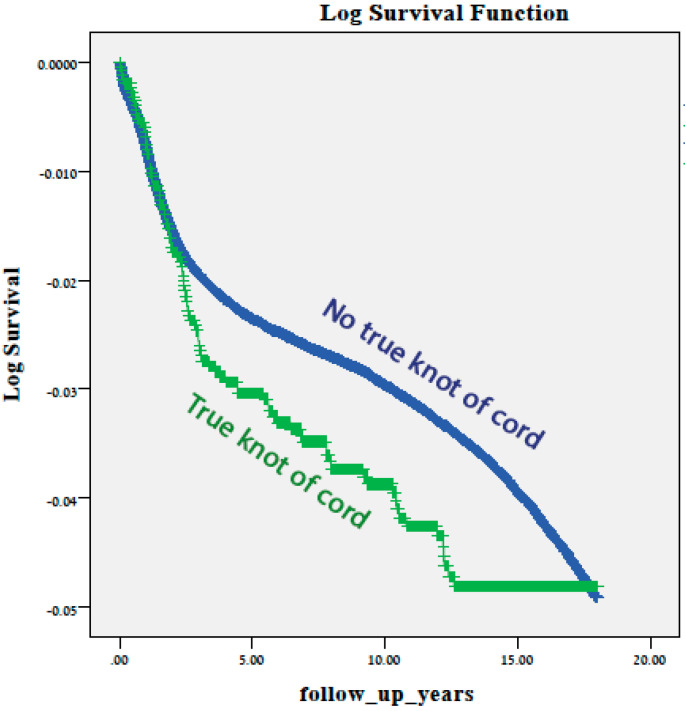
Log of survival, total neurological hospitalizations up to the age of 18 years by presence or absence of true knot of cord * (* log rank test *p* = 0.120).

For the long-term neurological morbidity analyses, perinatal mortality cases were excluded, leaving 242,342 newborns, 1.1% (2558) of which were exposed. During the 22 year follow up period (up to the age of 18), total neurological hospitalization rates were comparable between the groups (3.7% in the exposed group and 3.1% in the comparison group, *p* = 0.078; Table 3) as were the cumulative incidences of neurological hospitalizations over time (log rank *p* = 0.12; Figure 1). Attention deficit disorders associated with hospitalizations were slightly more common in the exposed group (0.16% vs. 0.06% in controls, *p* = 0.041).

The Cox regression model confirmed a lack of association between true knot of cord and total long term neurological related hospitalizations (adjusted HR = 1.236, 95% CI 0.728–2.1, *p* = 0.432; Table 4), as well as specifically for attention deficit disorders (adjusted HR 2.6, 95% CI 0.96–7.04, *p* = 0.06). The Cox model adjusted for diabetes, hypertensive disorders, maternal age and offspring date of birth. In a sensitivity analysis, the groups were stratified according to gestational age at delivery into term deliveries (37.0 weeks or more) and preterm deliveries (less than 37.0 weeks). The results remained similar (adjusted HR = 1.13, 95% CI 0.91–1.41, *p* = 0.261 for term deliveries and adjusted HR = 1.27, 95% CI 0.75–2.16, *p* = 0.365 for preterm deliveries). 

## 4. Discussion

In this large retrospective cohort study with a long follow up period, we found increased rates of adverse obstetrical outcomes in pregnancies associated with true knot of cord, and specifically with intra-uterine fetal demise, as well as low Apgar scores, preterm deliveries, cesarean deliveries, and meconium stained amniotic fluid. However, in the long term perspective, no association was found between true knot of cord and long term adverse neurological outcome (involving hospitalization) in the offspring, up to 18 years of age.

The increased rates of pretem delivery (PTD), cesarean delivery (CD), and low Apgar scores can potentially be explained by the association of true cord knots with non-reassuring fetal heart rate (NRFHR) and MSAF, thus predisposing these deliveries to iatrogenic interventions resulting in preterm deliveries, cesarean delivery [4,26] and low Apgar scores [27]. 

In addition, MSAF, polyhydramnios, true knot of cord and hypertensive disorders of pregnancy were all found to associated with IUFD [12], which can explain the significantly increased rate of IUFD in the exposed group. The association of true cord knots with IUFD appears to be significant and independent in the regression model, which was meticulously controlled for multiple confounders. In light of the severity of the immediate adverse outcomes reinforced by our study, it appears that increased antenatal surveillance is appropriate, in cases where a true knot of cord is diagnosed antenatally. It may also be appropriate to screen for it in high risk populations, if a reliable screening method was available. 

In contrast to the clear adverse impact of true knot exposure on perinatal outcome, our data conformed a lack of association between true cord knots and long-term neurological morbidity (associated with hospitalizations) in the offspring. To the best of our knowledge, no studies have previously focused on the long-term impact of true cord knots. We hypothesized that fetuses exposed to true knot of cord may have suffered some degree of hypoxemia during the pregnancy or labor process thus predisposing them to long term adverse neurological consequences. However, the results of this work suggest otherwise. True knot of cord may act in a severity dependent manner, meaning that the damage caused by the presence of the cord knot depends on the degree of venous flow obstruction caused by it, in a way that a tight knot may cause acute hypoxia, leading to immediate adverse outcome like IUFD; while a looser knot may result in chronic mild hypoxia and a less devastating outcome. In this manner, some or even most fetuses with knots might not be effected at all.

Several weaknesses of the study must be acknowledged:

1. Although several confounders were controlled for and an independent association was found with IUFD, it is possible due to the retrospective nature of the study, that some confounders were not accounted for.
2. Most childhood neurological morbidities, especially on the “lighter” side of the spectrum, are cared for in an ambulatory setting and were not accounted for in this long-term analysis. This can lead to under reporting of some diagnosis due to the fact that some diagnosed children are not hospitalized. Furthermore, for several of the outcomes (like autistic spectrum disorders), diagnosis typically only comes through specialized screening, which is a potential for selection bias (of children who suffer from the condition but were not screened for it). Nevertheless, some of the conditions included in the study are significant morbidities, and therefore are likely to necessitate hospitalization at some point. There is a possibility that the study groups were underpowered to detect neurological-related hospitalizations in the offspring.
3. Hospitalization at a different, distant, medical center, although unlikely, is possible. SUMC is the only tertiary center in the Negev region, it is reasonable to assume that this is the only place for children to be hospitalized in case of morbidity; however, there can be no guarantee of that. Therefore, ascertainment bias potentially exists. There seems to be no reason, however, for either of those phenomenon to be more common in either of the compared groups.
4. It was assumed that children that did not visit our hospital were healthy (which might be a biased assumption). This possibility as well is probably just as likely in both the exposed and unexposed groups.
5. A heterogeneous group of neurological outcomes was used rather than a specific neurological diagnosis. The purpose of this work was to search for an association between different groups of neurological morbidities and true knot of cord upon birth. We did not look for specific diagnoses since no specific associations were mentioned in the literature nor were part of our hypothesis. Additionally, these types of diagnoses are quite rare and looking for specific diagnoses (rather than groups of diagnoses) would have diminished the power of our results.

To conclude, the results of this large population based study with a long follow up period contribute some knowledge to the understating of the significance of true knot of cord. Although associated with elevated rates of IUFD, in our population, however, no severe long term neurological impact was noted.

## Figures and Tables

**Table 1:** Perinatal outcome according to presence or absence of true knot of the umbilical cord.

Perinatal Outcomes		True Knot of Cord % (*n* = 2606) *	No True Knot of Cord % (*n* = 241,076)	Odds Ratio (Confidence Interval)	*p-*Value	
Ethnicity	Jewish	60 (1572)	47.2 (113,782)			<0.001
	Bedouin	39.7 (1034)	52.8 (127,294)			<0.001
Mean maternal age (years, mean ± SD)		30.2 ± 5.9	28.1 ± 5.8			<0.001
Parity	1	15.5 (405)	23.7 (57,100)			<0.001
	2–4	54.8 (1428)	51.1 (123,086)			
	≥5	29.7 (773)	25.2 (60,837)			
Maternal Diabetes		8.4 (220)	5 (11,939)		1.77 (1.539–2.034)	<0.001
Maternal Hypertension		7.4 (192)	5 (12,055)		1.511 (1.303–1.752)	<0.001
Mean gestational age (weeks, mean ± SD **)		38.8 ± 2.4	39.1 ± 1.09			<0.001
Preterm delivery (<37 0/7 weeks of gestation)		10.5 (274)	6.8 (16,446)		1.605 (1.415–1.821)	<0.001
Induced labor		28.7 (747)	26.1 (62,897)		1.138 (1.045–1.24)	0.003
Cesarean delivery		17.4 (453)	13.5 (32,573)		1.347 (1.216–1.491)	<0.001
Placental abruption		0.8 (21)	0.6 (1338)		1.456 (0.944–2.244)	0.087
Meconium stained amniotic fluid		18.9 (493)	14.7 (35,399)		1.356 (1.228–1.496)	<0.001
Low (<7) 1 min Apgar score		7.3 (190)	5.3 (12,800)		1.403 (1.209–1.627)	<0.001
Low (<7) 5 min Apgar score		2.9 (76)	2.3 (5433)		1.303 (1.035–1.639)	0.024
Perinatal mortality	Total perinatal mortality	1.8 (48)	0.5 (1292)		3.483 (2.604–4.658)	<0.001
	Intra uterine	1.5 (39)	0.3 (713)		5.122 (3.702–7.086)	<0.001
	Intra-partum	0.1 (2)	0.024 (60)		3.085 (0.754–12.629)	0.099
	Immediately post-partum	0.3 (7)	0.2 (519)		1.248 (0.592–2.634)	0.560
Mean birth weight (grams, mean ± SD)		3209 ± 564	3205 ± 510			0.73
Low birth weight (<2500 g)		9.2 (239)	6.7 (16,165)		1.405 (1.229–1.606)	<0.001
Male gender		61.6 (1604)	50.7 (122,273)		1.555 (1.437–1.684)	<0.001
Female gender		38.4 (1002)	49.3 (118,803)		1.555 (1.437–1.684)	<0.001

* All numbers presented in % (*n*) unless otherwise stated, ** SD = Standard deviation.

**Table 2:** Multivariable regression analysis for the association between true knot of cord and perinatal mortality.

	Adjusted Odds Ratio (Confidence Interval)	*p-*Value
**True knot of cord**	3.606 (2.685–4.841)	<0.001
**Ethnicity (Jewish compared to Bedouin)**	0.595 (0.529–0.668)	<0.001
**Smoking**	1.52 (0.909–2.54)	0.11
**Maternal diabetes**	0.628 (0.463–0.852)	0.003
**Maternal Hypertension**	2.089 (1.733–2.518)	<0.001
**Birth year**	0.937 (0.929–0.946)	<0.001

**Table 3:** Long term neurological hospitalizations of the offspring born with and without true knot of the umbilical cord.

Neurological Morbidity	True Knot of Cord % (*n* = 2558)	No Knot of Cord % (*n* = 239,784)	*p-*Value
**Autistic spectrum disorders**	0.0003 (1)	0.0001 (27)	0.193
**Eating disorders**	0.2 (6)	0.2 (429)	0.508
**Sleeping disorders**	0.0003 (1)	0.0001 (47)	0.486
**Movement disorders**	2.2 (56)	1.8 (4416)	0.194
**Cerebral palsy**	0.1 (2)	0.1 (199)	0.933
**Psychiatric emotional**	0.5 (12)	0.5 (1183)	0.862
**Attention deficit disorders**	0.2 (4)	0.1 (139)	0.041
**Developmental disorders**	0.2 (5)	0.1 (234)	0.117
**Degenerative, demyelination**	0.03 (1)	0.1 (180)	0.508
**Headache**	0 (0)	0.0002 (54)	0.448
**Myopathy**	0.1 (2)	0.1 (136)	0.651
**Other**	0.4 (10)	0.4 (907)	0.917
**Total Neurological hospitalizations**	3.7 (95)	3.1 (7448)	0.078

**Table 4:** Cox regression analysis for the association between long term neurological morbidity and true knot of cord.

	Adjusted Hazard Ratio (Confidence Interval)	*p* Value
**True knot of cord**	1.236 (0.728–2.1)	0.432
**Diabetes**	1.143 (0.87–1.501)	0.337
**Hypertension**	1.249 (1.017–1.534)	0.034
**Maternal age (at birth)**	0.993 (0.981–1.005)	0.265
**Child birth year**	1.092 (1.076–1.109)	<0.001

## Data Availability

According to the local Helsinky guidelines data cannot be provided outside of hospital.

## Appendix A

ICD-9 codes for neurologic diagnosis.

**Table A1:** Supplement A-Table—List of Neurological Diagnoses.

Group	Subgroup	Code	Diagnosis Description
Neurology	Autistic spectrum disorders	2990	Autistic Disorder
		2990	Infantile Autism
		29,900	Autistic Disorder, Current or Active State
		29,901	Autistic Disorder, Residual State
		29,910	Childhood Disintegrative Disorder, Current or Active State
		2998	Other Specified Pervasive Developmental Disorders
		29,981	Other Specified Pervasive Developmental Disorders, Residula State
		29,990	Unspecif. Pervasive Developmental Disorder, Current or Active State
	Eating disorders	3071	Anorexia Nervosa
		3075	Other and Unspecified Disorders of Eating
		30,750	Eating Disorder, Unspecified
		30,751	Bulimia Nervosa
		30,753	Rumination Disorder
		30,759	Other Disorders of Eating
		V691	Inappropriate Diet & Eating Habits
	Sleeping disorders	3073	Stereotypic Movement Disorder
		30,746	Sleep Arousal Disorder
		30,746	Somnambulism or Night Terrors
		30,747	Other Dysfunctions of Sleep Stages or Arousal from Sleep
		32,727	Central Sleep Apnea in Conditions Classified Elsewhere
		32,730	Circadian Rhythm Sleep Disorder, Unspecified
		32,732	Circadian Rhythm Sleep Disorder, Advanced Sleep Phase Type
		34,700	Narcolepsy without Cataplexy
		34,701	Narcolepsy with Cataplexy
		7805	Sleep Disturbances
		78,050	Unspecified Sleep Disturbance
		78,051	Insomnia with Sleep Apnea
		78,051	Insomnia with Sleep Apnea, Unspecified
		78,052	Insomnia, Unspecified
		78,052	Other Insomnia
		78,054	Hypersomnia, Unspecified
		78,056	Dysfunctions Associated with Sleep Stages or Arousal from Sleep
		78,059	Other Sleep Disturbances
		V694	Lack of Adequate Sleep
	Movement disorders	3331	Essential and Other Specified Forms of Tremor
		3332	Myoclonus
		3335	Other Choreas
		3336	Genetic Torsion Dystonia
		3336	Idiopathic Torsion Dystonia
		33,390	Unsp. Extrapyramidal Disease + Abnormal Movement Disorder
		33,399	Other Extrapyramidal Diseases and Abnormal Movement Disorders
		3343	Other Cerebellar Ataxia
	Epilepsy	3450	Generalized Nonconvulsive Epilepsy
		34,500	Generalized Nonconvulsive Epilepsy without Intractable Epilepsy
		34,501	Generalized Nonconvulsive Epilepsy with Intractable Epilepsy
		34,510	Generalized Convulsive Epilepsy without Intractable Epilepsy
		34,511	Generalized Convulsive Epilepsy with Intractable Epilepsy
		3452	Petit Mal Status, Epileptic
		3453	Grand Mal Status, Epileptic
		34,540	Partial Epilepsy + Impairment of Consciousness without Intractable Epilepsy
		3455	Partial Epilepsy, without Impairment of Consciousness
		34,550	Partial Epilepsy without Impairment of Consciousness without Intr Actabel Epilepsy
		3456	Infantile Spasms
		34,560	Infantile Spasms without Intractable Epilepsy
		3459	Epilepsy, Unspecified
		34,590	Epilepsy, Nusp. without Intractabel Epilepsy
		34,590	Epilepsy, Unsp. without Intractable Epilepsy
		34,591	Epilepsy Unsp. With Intractable Epilepsy
		78,039	Other Convulsions
		7810	Abnormal Involuntary Movements
		7812	Abnormality of Gait
		7813	Lack of Coordination
	Cerebral palsy	3341	Hereditary Spastic Paraplegia
		3421	Spastic Hemiplegia
		34,210	Spastic Hemiplegia Affecting Unsp. Side
		3429	Hemiplegia, Unspecified
		34,290	Hemiplegia, Unsp., Affecting Unsp. Side
		34,291	Hemiplegia, Unsp., Affecting Dominant Side
		34,292	Hemiplegia, Unsp., Affecting Nondominant Side
		3430	Congenital Diplegia
		3431	Congenital Hemiplegia
		3432	Congenital Quadriplegia
		3439	Infantile Cerebral Palsy, Unspecified
		34,400	Quadriplegia, Unspecified
		3441	Paraplegia
		3442	Diplegia Of Upper Limbs
		34,430	Monoplegia of Lower Limb, Affecting Unsp. Side
		34,440	Monoplegia of Upper Limb, Affecting Unsp. Side
		34,489	Other Specified Paralytic Syndrome
		3449	Paralysis, Unspecified
		3481	Anoxic Brain Damage
		3526	Multiple Cranial Nerve Palsies
		43,811	Aphasia
		43,820	Hemiplegia Affecting Unsp. Side
		7814	Transient Paralysis of Limb
	Psychiatric disorders	2930	Acute Delirium
		2930	Delirium Due to Conditions Classified Elsewhere
		29,384	Anxiety Disorder in Conditions Classified Elsewhere
		2940	Amnestic Disorder in Conditions Classified Elsewhere
		2949	Unspecified Persistent Mental Disorders Due to Cond. Class. Elsewh.
		29,530	Paranoid Type Schizophrenia, Unspecified State
		29,570	Schizoaffective Disorder Schizophrenia, Unspecified State
		29,580	Other Specified Types of Schizophrenia, Unspecified State
		29,590	Unspecified Type Schizophrenia, Unspecified State
		29,600	Bipolar I Disorder, Single Manic Episode, Unspecified Degree
		29,620	Major Depressive Affective Disorder, Single Episode, Unsp. Degree
		29,680	Bipolar Disorder, Unspecified
		29,690	Unspecified Episodic Mood Disorder
		29,699	Other Specified Affective Psychoses
		2971	Delusional Disorder
		2979	Unspecified Paranoid State
		2981	Excitative Type Psychosis
		2983	Acute Paranoid Reaction
		2989	Unspecified Psychosis
		30,000	Anxiety State, Unspecified
		30,001	Panic Disorder without Agoraphobia
		30,009	Other Anxiety States
		30,010	Hysteria, Unspecified
		30,011	Conversion Disorder
		30,029	Other Isolated or Simple Phobias
		3003	Obsessive-Compulsive Disorders
		3004	Dysthymic Disorder
		3004	Neurotic Depression
		3009	Unspecified Nonpsychotic Mental Disorder
		30,183	Borderline Personality
		30,183	Borderline Personality Disorder
		3019	Unspecified Personality Disorder
		3026	Disorders of Psychosexual Identity
		30,302	Ac. Alcoholic Intoxic. in Alcoholism, Episodic Drinking Behavior
		30,400	Opioid Type Dependence, Unspecified Use
		30,430	Cannabis Dependence, Unspecified Use
		30,432	Cannabis Dependence, Episodic Use
		30,500	Alcohol Abuse, Unspecified Drinking Behavior
		30,501	Alcohol Abuse, Continuous Drinking Behavior
		30,502	Alcohol Abuse, Episodic Drinking Behavior
		3051	Tobacco Use Disorder (Tobacco Dependence)
		30,591	Other, Mixed, Or Unspecified Drug Abuse, Continuous Use
		3061	Respiratory Malfunction Arising from Mental Factors
		3062	Cardiovascular Malfunction Arising from Mental Factors
		3068	Other Specified Psychophysiological Malfunction
		3069	Unspecified Psychophysiological Malfunction
		3070	Adult Onset Fluency Disorder
		3070	Stammering and Stuttering
		3070	Stuttering
		30,720	Tic Disorder, Unspecified
		30,722	Chronic Motor or Vocal Tic Disorder
		30,723	Tourette’s Disorder
		30,752	Pica
		3080	Predominant Disturbance of Emotions
		3089	Unspecified Acute Reaction to Stress
		309	Adjustment Reaction
		3090	Adjustment Disorder with Depressed Mood
		30,924	Adjustment Disorder with Anxiety
		3094	Adjustment Disor. with Mixed Disturb. of Emotions and Conduct
		30,981	Posttraumatic Stress Disorder
		3099	Unspecified Adjustment Reaction
		311	Depressive Disorder, Not Elsewhere Classified
		31,210	Undersocialized Conduct Disorder, Unaggressive Type, Unspecified
		31,239	Other Disorders of Impulse Control
		3129	Unspecified Disturbance of Conduct
		31,389	Other Emotional Disturbances of Childhood or Adolescence
		3139	Unspecified Emotional Disturbance of Childhood or Adolescence
		316	Psychic Factors Associated with Diseases Classified Elsewhere
		7801	Hallucinations
		7803	Convulsions
		7992	Nervousness
		79,921	Nervousness
		79,922	Irritability
		79,925	Demoralization and Apathy
		79,929	Other Signs and Symptoms Involving Emotional State
		7993	Debility, Unspecified
		V6284	Suicidal Ideation
	Attention deficit disorders	31,400	Attention Deficit Disorder without Hyperactivity
		31,401	Attention Deficit Disorder with Hyperactivity
		3142	Hyperkinetic Conduct Disorder of Childhood
		3149	Unspecified Hyperkinetic Syndrome of Childhood
		V400	Mental and Behavioral Problems with Learning
		V409	Unspecified Mental or Behavioral Problem
	Developmental disorders	3152	Other Specific Developmental Learning Difficulties
		31,531	Expressive Language Disorder
		31,534	Speech and Language Developmental Delay Due to Hearing Loss
		31,539	Other Developmental Speech Disorder
		3154	Developmental Coordination Disorder
		3158	Other Specified Delays in Development
		3159	Unspecified Delay in Development
		317	Mild Intellecutal Disabilities
		317	Mild Mental Retardation
		319	Unspecified Intellectual Disabilities
		319	Unspecified Mental Retardation
		33,183	Mild Cognitive Impairment, So Stated
		7834	Lack of Expected Normal Physiological Development
		7834	Lack of Expected Normal Physiological Development in Childhood
		78,340	Lack of Normal Physiological Development, Unspecified
	Degenerative disorders	330	Cerebral Degenerations Usually Manifest in Childhood
		3300	Leukodystrophy
		3308	Other Specified Cerebral Degenerations in Childhood
		3313	Communicating Hydrocephalus
		33,132	Post Hemorrhagic Hydrocephalus
		3314	Obstructive Hydrocephalus
		33,189	Other Cerebral Degeneration
		3319	Cerebral Degeneration, Unspecified
		3348	Other Spinocerebellar Diseases
		335	Anterior Horn Cell Disease
		3350	Werdnig-Hoffmann Disease
		33,510	Spinal Muscular Atrophy, Unspecified
		33,522	Progressive Bulbar Palsy
		33,523	Pseudobulbar Palsy
		3360	Syringomyelia And Syringobulbia
		340	Multiple Sclerosis
		3410	Neuromyelitis Optica
		3411	Schilder’s Disease
		34,120	Acute (Transverse) Myelitis Nos
		3419	Demyelinating Disease of Central Nervous System, Unspecified
		3480	Cerebral Cysts
		348,891	Cerebral Calcification
		3590	Congenital Hereditary Muscular Dystrophy
		3591	Hereditary Progressive Muscular Dystrophy
	Headache	30,781	Tension Headache
		34,600	Migraine With Aura without Mention Of Intractable Migraine, Without Mention Of Status Migrainosus
		34,601	Migraine with Aura, So Stated, without Mention of Statu. Migrainosus
		34,620	Variants of Migraine, without Intractable Migraine
		34,630	Hemiplegic Migraine without Mention of Intractable Migraine, With Out Mention of Status Migrainosus
		34,670	Chronic Migraine without Aura without Mention of Intractable Migr Aine, without Mention of Status Migrainosus
		3469	Migraine, Unspecified
		34,690	Migraine, Unspecified, without Intractabel Migraine
		34,690	Migraine, Unspecified, without Mention of Intractable Migraine Wi Thout Mention of Status Migrainosus
	Myopathy	3556	Lesion of Plantar Nerve
		33,709	Other Idiopathic Peripheral Autonomic Neuropathy
		33,720	Reflex Sympathetic Dystrophy, Unspecified
		33,721	Reflex Sympathetic Dystrophy of Upper Limb
		33,722	Reflex Sympathetic Dystrophy of Lower Limb
		3379	Unspecified Disorder of Autonomic Nervous System
		3510	Bell’s Palsy
		3518	Other Facial Nerve Disorders
		3519	Facial Nerve Disorder, Unspecified
		352	Disorders of Other Cranial Nerves
		3539	Unspecified Nerve Root and Plexus Disorder
		3542	Lesion of Ulnar Nerve
		3548	Other Mononeuritis of Upper Limb
		3549	Mononeuritis of Upper Limb, Unspecified
		3553	Lesion of Lateral Popliteal Nerve
		3558	Mononeuritis of Lower Limb, Unspecified
		3559	Mononeuritis of Unspecified Site
		3562	Hereditary Sensory Neuropathy
		3564	Idiopathic Progressive Polyneuropathy
		3568	Other Specified Idiopathic Peripheral Neuropathy
		3569	Unspecified Idiopathic Peripheral Neuropathy
		3570	Acute Infective Polyneuritis
		3571	Polyneuropathy in Collagen Vascular Disease
		3572	Polyneuropathy in Diabetes
		3577	Polyneuropathy Due to Other Toxic Agents
		35,781	Chronic Inflammatory Demyelinating Polyneuritis
		35,800	Myasthenia Gravis without (Acute) Exacerbation
		3588	Other Specified Myoneural Disorders
		3589	Myoneural Disorders, Unspecified
		3592	Myotonic Disorders
		3599	Myopathy, Unspecified
	Others	30,789	Other Psychalgia
		33,381	Blepharospasm
		3384	Chronic Pain Syndrome
		33,903	Episodic Paroxysmal Hemicrania
		3482	Benign Intracranial Hypertension
		3483	Encephalopathy, Unspecified
		3483	Encephalopathy, not Elsewhere Classified
		34,830	Encephalopathy, Unspecified
		34,831	Metabolic Encephalopathy
		34,881	Cerebral Calcification
		34,881	Temporal Sclerosis
		34,889	Other Conditions of Brain
		3490	Reaction to Spinal or Lumbar Puncture
		3492	Disorders of Meninges, not Elsewhere Classified
		34,981	Cerebrospinal Fluid Rhinorrhea
		34,989	Other Specified Disorders of Nervous System
		3499	Unspecified Disorders of Nervous System
		3561	Peroneal Muscular Atrophy
		7802	Syncope and Collapse
		78,093	Memory Loss
		7843	Aphasia
		99,701	Central Nervous System Complication
		99,709	Other Nervous System Complications

## References

[1] Spellacy W.N., Gravem H., Fisch R.O. (1966). The umbilical cord complications of true knots, nuchal coils, and cords around the body. Report from the collaborative study of cerebral palsy. Am. J. Obstet. Gynecol..

[2] Sepulveda W., Shennan A.H., Bower S., Nicolaidis P., Fisk N.M. (1995). True knot of the umbilical cord: A difficult prenatal ultrasonographic diagnosis. Ultrasound Obstet. Gynecol..

[3] Weintraub A.Y., Sheiner E., Bashiri A., Shoham-Vardi I., Mazor M. (2005). Is there a higher prevalence of pregnancy complications in a live-birth preceding the appearance of recurrent abortions?. Arch. Gynecol. Obstet..

[4] Hershkovitz R., Silberstein T., Sheiner E., Shoham-Vardi I., Holcberg G., Katz M., Mazor M. (2001). Risk factors associated with true knots of the umbilical cord. Eur. J. Obstet. Gynecol. Reprod. Biol..

[5] Maymon E., Ghezzi F., Shoham-Vardi I., Franchi M., Silberstein T., Wiznitzer A., Mazor M. (1998). Isolated hydramnios at term gestation and the occurrence of peripartum complications. Eur. J. Obstet. Gynecol. Reprod. Biol..

[6] Blickstein I., Shoham-Schwartz Z., Lancet M. (1987). Predisposing factors in the formation of true knots of the umbilical cord—Analysis of morphometric and perinatal data. Int. J. Gynaecol. Obstet..

[7] Didion S.P., Kinzenbaw D.A., Schrader L.I., Chu Y., Faraci F.M. (2009). Endogenous interleukin-10 inhibits angiotensin II-induced vascular dysfunction. Hypertension.

[8] Maher J.T., Conti J.A. (1996). A comparison of umbilical cord blood gas values between newborns with and without true knots. Obstet. Gynecol..

[9] Jauniaux E., Campbell S., Vyas S. (1989). The use of color Doppler imaging for prenatal diagnosis of umbilical cord anomalies: Report of three cases. Am. J. Obstet. Gynecol..

[10] Gutvirtz G., Walfisch A., Beharier O., Sheiner E. (2016). Isolated single umbilical artery is an independent risk factor for perinatal mortality and adverse outcomes in term neonates. Arch. Gynecol. Obstet..

[11] Airas U., Heinonen S. (2002). Clinical Significance of True Umbilical Knots: A Population-Based Analysis. Am. J. Perinatol..

[12] Ohana O., Holcberg G., Sergienko R., Sheiner E. (2011). Risk factors for intrauterine fetal death (1988–2009). J. Matern. Neonatal Med..

[13] Matorras R., Diez J., Pereira J.G., Montoya F., de Terán G.G., Aranguren G., Rodriguez-Escudero F.J. (1990). True knots in the umbilical cord: Clinical findings and fetal consequences. J. Obstet. Gynaecol..

[14] McLennan H., Price E., Urbanska M., Craig N., Fraser M. (1988). Umbilical cord knots and encirclements. Aust. N. Z. J. Obstet. Gynaecol..

[15] Räisänen S., Georgiadis L., Harju M., Keski-Nisula L., Heinonen S. (2013). True umbilical cord knot and obstetric outcome. Int. J. Gynecol. Obstet..

[16] Lackman F., Capewell V., Gagnon R., Richardson B. (2001). Fetal umbilical cord oxygen values and birth to placental weight ratio in relation to size at birth. Am. J. Obstet. Gynecol..

[17] Chasnoff I.J., Fletcher M.A. (1977). True knot of the umbilical cord. Am. J. Obstet. Gynecol..

[18] Estis-Deaton A., Sheiner E., Wainstock T., Landau D., Walfisch A. (2017). The association between inadequate prenatal care and future healthcare use among offspring in the Bedouin population. Int. J. Gynaecol. Obstet..

[19] Abu-Ghanem S., Sheiner E., Sherf M., Wiznitzer A., Sergienko R., Shoham-Vardi I. (2012). Lack of prenatal care in a traditional community: Trends and perinatal outcomes. Arch. Gynecol. Obstet..

[20] Sheiner E., Shoham-Vardi I., Weitzman D., Gohar J., Carmi R. (1998). Decisions regarding pregnancy termination among Bedouin couples referred to third level ultrasound clinic. Eur. J. Obstet. Gynecol. Reprod. Biol..

[21] Sheiner E., Shoham-Vardi I., Ohana E., Segal D., Mazor M., Katz M. (1999). Characteristics of parturients who choose to deliver without analgesia. J. Psychosom. Obstet. Gynaecol..

[22] Amir H., Abokaf H., Levy Y.A., Azem F., Sheiner E. (2018). Bedouin Women’s Gender Preferences When Choosing Obstetricians and Gynecologists. J. Immigr. Minority Health.

[23] Treister-Goltzman Y., Peleg R. (2014). Health and Morbidity Among Bedouin Women in Southern Israel: A Descriptive Literature Review of the Past Two Decades. Journal of Community Health.

[24] Lewando-Hundt G., Shoham-Vardi I., Beckerleg S., Belmaker I., Kassem F., Jaafar A.A. (2001). Knowledge, action and resistance: The selective use of pre-natal screening among Bedouin women of the Negev, Israel. Soc. Sci. Med..

[25] Pariente G., Wainstock T., Walfisch A., Landau D., Sheiner E. (2019). Placental abruption and long-term neurological hospitalisations in the offspring. Paediatr. Perinat. Epidemiol..

[26] Weiner E., Fainstein N., Schreiber L., Sagiv R., Bar J., Kovo M. (2015). The association between umbilical cord abnormalities and the development of non-reassuring fetal heart rate leading to emergent cesarean deliveries. J. Perinatol..

[27] Lai S., Flatley C., Kumar S. (2017). Perinatal risk factors for low and moderate five-minute Apgar scores at term. Eur. J. Obstet. Gynecol. Reprod. Biol..

